# Accuracy and precision of depth-resolved estimation of attenuation coefficients in optical coherence tomography

**DOI:** 10.1117/1.JBO.28.6.066001

**Published:** 2023-06-14

**Authors:** Linda B. Neubrand, Ton G. van Leeuwen, Dirk J. Faber

**Affiliations:** a Amsterdam UMC, Location University of Amsterdam, Department of Biomedical Engineering and Physics, Amsterdam, The Netherlands; b Amsterdam Cardiovascular Sciences, Atherosclerosis and Ischemic Syndromes, Amsterdam, The Netherlands; c Cancer Center Amsterdam, Imaging and Biomarkers, Amsterdam, The Netherlands

**Keywords:** optical coherence tomography, attenuation coefficient, Cramér–Rao lower bound, depth resolved estimation, curve-fitting, OCT signal simulation

## Abstract

**Significance:**

Parametric imaging of the attenuation coefficient $\mu_{\mathrm{OCT}}$ using optical coherence tomography (OCT) is a promising approach for evaluating abnormalities in tissue. To date, a standardized measure of accuracy and precision of $\mu_{\mathrm{OCT}}$ by the depth-resolved estimation (DRE) method, as an alternative to least squares fitting, is missing.

**Aim:**

We present a robust theoretical framework to determine accuracy and precision of the DRE of $\mu_{\mathrm{OCT}}$.

**Approach:**

We derive and validate analytical expressions for the accuracy and precision of $\mu_{\mathrm{OCT}}$ determination by the DRE using simulated OCT signals in absence and presence of noise. We compare the theoretically achievable precisions of the DRE method and the least-squares fitting approach.

**Results:**

Our analytical expressions agree with the numerical simulations for high signal-to-noise ratios and qualitatively describe the dependence on noise otherwise. A commonly used simplification of the DRE method results in a systematic overestimation of the attenuation coefficient in the order of $\mu_{\mathrm{OCT}}^{2}\times \Delta$, where Δ is the pixel stepsize. When $\mu_{\mathrm{OCT}}\cdot |\mathrm{AFR}|≲1.8$, $\mu_{\mathrm{OCT}}$ is reconstructed with higher precision by the depth-resolved method compared to fitting over the length of an axial fitting range |AFR|.

**Conclusions:**

We derived and validated expressions for the accuracy and precision of DRE of $\mu_{\mathrm{OCT}}$. A commonly used simplification of this method is not recommended as being used for OCT-attenuation reconstruction. We give a rule of thumb providing guidance in the choice of estimation method.

## 1 Introduction

Genesis and progression of disease are accompanied by morphological changes in tissues on length scales ranging from intracellular organelles to macroscopic tissue structures. These lead to changes in the spatial distribution of the complex refractive index, which in turn leads to changes in the absorption and scattering properties that can be measured using optical techniques. The main hypothesis underlying many applications of biophotonics is that, by measuring the optical properties, diagnosis or monitoring of tissue disease state or treatment is possible. Consequently, knowledge of the accuracy and precision of the methods to assess these optical properties is paramount.

The optical property accessible with optical coherence tomography (OCT) measurements is the attenuation coefficient, which describes the decay rate of the OCT signal with depth.^1^^,^^2^ It is commonly extracted by non-linear least squares curve fitting (CF) of a single scattering-based model to the OCT signal.^3^ The main cause of imprecision in the determination of the attenuation coefficient is the inherent random fluctuation of the OCT signal, due to speckle and (shot) noise. Speckle is the voxel-to-voxel variation of OCT amplitude, caused by the spatial variation of the refractive index in tissue.^4^^,^^5^ Randomly placed scatterers within the voxels will return scattered fields with random amplitude and phase, leading to intensity fluctuations at the detector.^6^ We have recently derived a simple expression for the minimal attainable precision with which the attenuation coefficient can be determined using CF based on the so-called Cramér–Rao (CR) lower bound:^7^

$$\sigma_{\mu_{\mathrm{OCT},\mathrm{CF}}}=\frac{1}{\left|\mathrm{AFR}\right|}\sqrt{\frac{3c_{R}}{MN}},$$ (1)

where |AFR| is the length of the axial fitting range (AFR), M is the number of independent data points in the AFR, and N is the number of A-scans averaged prior to fitting. The constant $c_{R}=4(4-\pi )/\pi$ originates from the Rayleigh distribution of amplitude values corresponding to fully developed speckle. The lower bound given by Eq. (1) is expressed in the same units as the attenuation coefficient, e.g., ${\mathrm{mm}}^{-1}$ and corresponds to the standard deviation of the normal distribution of attenuation coefficients that would be obtained by repeating the fitting procedure a large number of times, each time with a different, random, realization of the speckle pattern. The main feature of Eq. (1) is that the precision is independent of the attenuation coefficient itself but only depends on the parameters used in the fitting procedure. In the derivation of Eq. (1), we assumed that shot noise was negligible. Our results validated this assumption when signals-to-noise ratios (SNRs) within the AFR exceed 20 dB.^7^

CF suffers from the drawback, as can be inferred from Eq. (1), that a finite sized AFR is necessary to achieve sufficient precision, which may preclude measurement of the attenuation coefficient of thin layers, such as in the retina or the arterial wall, or regions near the basal membrane.^8^ In recent years, the depth-resolved estimation (DRE) method has grown popular as an alternative to CF. Introduced to the OCT field by Vermeer et al.,^9^ it was inspired by earlier work on shadow removal in OCT^10^ and on ultrasound attenuation compensation,^11^ once again demonstrating one of many conceptual similarities between both modalities. Practical improvements of the method were introduced by Liu et al.,^12^ Smith et al.,^13^ and Dwork et al.^14^ The principal allure of the method is the (apparent) pixel-wise determination of the attenuation coefficient, which may circumvent the need for an AFR that extends far into depth.^15^ However, assessment of the accuracy and precision of DRE estimation is scarce. The aim of this study therefore is to determine the accuracy and precision of the DRE of the attenuation coefficient.

## 2 Theory

Under the assumption of single backscattering from a homogeneous medium with stationary optical properties, the OCT signal versus depth z is modeled as a single exponential decay combined with the confocal point spread function and sensitivity roll-off:^2^

$${\left\langle i_{d}(z)\right\rangle }^{2}=\alpha T(z-z_{f})\cdot H(z-z_{0})\cdot \mu_{b,\mathrm{NA}}\exp(-2\mu_{\mathrm{OCT}}z)+{\left\langle \zeta \right\rangle }^{2},$$ (2)

where α is a conversion factor that includes the detector response, T(z) is the confocal point spread function, $H(z-z_{0})$ describes the sensitivity roll-off in depth for non-time domain OCT, $\mu_{b,\mathrm{NA}}$ is the backscattering coefficient within the numerical aperture (NA) of the detection optics; $\mu_{\mathrm{OCT}}$ is the OCT attenuation coefficient that contains contributions from both scattering and absorption. In the absence of multiple forward scattering, which we will assume henceforth, $\mu_{\mathrm{OCT}}=\mu_{s}+\mu_{a}$ is the sum of scattering and absorption coefficients. The backscattering coefficient is proportional to the scattering coefficient $\mu_{s}$ through a phase function and NA dependent factor $p_{\mathrm{NA}}$. The mean squared noise background is given by ${\left\langle \zeta \right\rangle }^{2}$. Upon noise subtraction and following correction for point spread function and roll-off, we arrive at

$$I(z)\propto p_{\mathrm{NA}}\mu_{s}\;\exp(-2\mu_{\mathrm{OCT}}z).$$ (3)

We proceed to compute the definite integral $I_{E}(z)=\int_{z}^{E}I(z^{\prime })dz^{\prime }$ of Eq. (3), which runs from the depth z up to the end of the available (or used) data range E, to estimate the attenuation coefficient as (Appendix A):

μ^OCT(z)=I(z)2IE(z)+I(E)/μ^E. (4)

Compared to the original formulation by Vermeer, Eq. (4) contains a regularization term I(E)/μ^E in the denominator to compensate for the finite data range.^16^ Here, μ^E=μ^OCT(E) is an independently obtained estimate for the attenuation coefficient at the end of the data range E, which may be found, e.g., by CF or from transmission measurements.^17^ Due to speckle and noise fluctuations, the OCT signal is itself an inherently fluctuating quantity of which Eq. (2) represents the average. Inspection of Eq. (4) suggests that these fluctuations will be largely averaged out only in the denominator I(z) due to the integration. Indeed, as shown by Fiske et al.,^18^ the attenuation coefficient retrieved by the DRE follows the same statistical distribution as the OCT intensity I(z), which is a Rayleigh distribution when the OCT signal is represented on amplitude basis, or an exponential distribution when the OCT signal is represented on an intensity basis as in this article:

p(μ^OCT)=1⟨μ^OCT⟩ exp(−μ^OCT/⟨μ^OCT)⟩. (5)

The mean value ⟨μ^OCT⟩ can be obtained from a large set of estimations of μ^OCT(z), in practice over some spatial range around z and/or from several A-scans at the same position. Combining Eqs. (3) and (4), we theoretically obtain

⟨μ^OCT(z)⟩=μOCT×11−[1−μOCTμ^E]exp(−2μ^OCT((E−z)). (6)

Thus, ⟨μ^OCT(z)⟩→μOCT at a location sufficiently far from E, whereas ⟨μ^OCT(z)→μ^OCT(E)⟩ as z approaches E. Vermeer considered the effect of discretization of I(z), i.e., each datapoint I[i] corresponds to the integration of Eq. (3) over a finite pixel size Δ around z. The exact, discretized version of Eq. (4) reads (Appendix B)

μ^OCT[i]=12Δ ln(1+I[i]∑j=i+1imaxI[j]+C), (7)

where $i_{\max}=E/\Delta$ is the pixel index corresponding to the end of the data range. The factor C=I[imax]/(exp(2μ^EΔ)−1) is the discretized equivalent of the term I(E)/μ^E in Eq. (4).

Often, a simplified version of Eq. (7) is used by linearization of the logarithmic and exponential terms [perhaps inspired by the closer visual resemblance to Eq. (4)]

μ^OCT[i]=I[i]2Δ∑j=i+1imaxI[j]+CL. (8)

Moreover, some authors further omit the (linearized) regularization term CL=I[imax]/μ^OCT[imax] from Eq. (8). Use of these approximations is discouraged as they come with the penalty of reduced accuracy. The analysis in Appendix B reveals that Eq. (8) systematically overestimates the attenuation coefficient in the order of $\mu_{\mathrm{OCT}}^{2}\times \Delta$.

We now seek the precision with which ⟨μ^OCT[i]⟩ can be estimated with maximum likelihood (ML) from the Fisher information associated with the data using a CR analysis. Conceptually, Fisher information measures the amount of information that a dataset provides about the parameters of a model for the data. The CR lower bound, the inverse of Fisher information, measures the highest precision with which the parameters can be estimated using ML methods. In the case of CF of OCT intensity (or amplitude) values, the parameter of interest is the attenuation coefficient. In the present case, somewhat trivially, the parameter of interest is the mean of the set of μ^OCT -estimations obtained by Eq. (7), which are distributed according to Eq. (5). The ML estimator of the mean of an exponential distribution is simply the arithmetic mean of the estimations. The Fisher information for M independent estimations from an exponential distribution is Ϝexp=M/⟨μ^OCT⟩2 so the CR lower bound, expressed as standard deviation becomes σμOCT,DRE=⟨μ^OCT⟩/M. Commonly, A-scans are pre-averaged prior to the application of the depth-resolved estimation. This changes the distribution of recovered attenuation coefficients to an approximately normal distributions when the number of over averages is N≳30, with mean equal to ⟨μ^OCT⟩ and variance equal to σN2=⟨μ^OCT⟩2/N. The corresponding Fisher information for the estimated attenuation coefficient based on M independent measurements then becomes Ϝ=MN/⟨μ^OCT⟩2 and the CR lower bound, expressed as standard deviation is thus σμOCT,DRE=⟨μ^OCT⟩/MN. From a frequentist statistician’s point of view, this quantity represents the standard deviation of the normal distribution of attenuation coefficient values, which would be obtained if the DRE analysis were repeated many times. We can use this interpretation to calculate the decrease in precision under the influence of noise. The result of the lower bound of this analysis, which can be found in Appendix C, is

σμ^OCT,DRE(i)=⟨μ^OCT[i]⟩MN·1+1SNR[i]2, (9)

where the SNR is defined per pixel as SNR[i]=I[i]/⟨ζ⟩.

Comparing Eq. (9) to Eq. (1), we see that the precision in the DRE estimation method is directly proportional to the pixel-wise estimate of the attenuation coefficient, whereas it is independent of ⟨μ^OCT⟩ for CF. It also shows that a higher precision can be obtained using DRE compared to CF, when the AFR becomes smaller than 3cR/μ^OCT or roughly two mean free paths.

## 3 Methods

To validate the accuracy and precision derived in Eqs. (6) and (9), we performed numerical simulations based on OCT scans from a homogeneous medium. Details of our simulation procedure can be found in Ref. 7. Briefly, single A-scans with randomly varying amplitude were generated based on $A_{\mathrm{sim}}(z_{i})=\sqrt{-\frac{4}{4-\pi }\cdot \sigma_{A}^{2}(z_{i})\cdot \ln(\xi_{i})}$, where $\sigma_{A}^{2}(z)=\frac{c_{R}}{4}(I(z)+{\left\langle \zeta \right\rangle }^{2})$ is the amplitude variance, I(z) is given by Eq. (3), and $\xi_{i}$ is a uniformly distributed number between 0 and 1 drawn for each pixel. This procedure assures that the amplitudes $A_{\mathrm{sim}}(z)$ follow a Rayleigh distribution corresponding to fully developed speckle and the contribution of shot noise. N=100 of these A-scans are first squared, then averaged so the resulting averaged intensities at each depth position are normally distributed. Thereafter, we subtract the mean noise floor ⟨ζ⟩.

Each squared, averaged A-scan is processed using Eq. (7) to estimate the attenuation coefficient. To demonstrate the spurious effect of linearization, we also analyzed the data using Eq. (8) with the regularization term $C_{L}$ omitted. This procedure was repeated $10^{4}$ times to obtain a distribution of μ^OCT[i] estimations at each depth position. Comparing the mean of this distribution to the input attenuation coefficient yields the accuracy of the method, and the precision is given by the distribution’s standard deviation.

We used comparable simulation parameters as previously^7^ reported for a direct comparison between the precisions of least squared fitting and the DRE method used in this article. In the simulations, $p_{\mathrm{NA}}$ was set to unity and an arbitrary scaling factor of $2500^{2}$ was included. Simulations were performed both with and without shot noise included. In the latter case, signal fluctuations are caused only by speckle. In the former case, the mean noise level was fixed at ζ=13.5. Values of 2 and $5\;{\mathrm{mm}}^{-1}$ were used for the attenuation coefficient, which leads to a maximum SNR expressed in decibels of 60 and 64 dB, respectively. In all simulations, we used a value of μ^OCT(E)=5  mm−1 for the estimation of the attenuation coefficient at the end of the data range E.

## 4 Results

**Fig. 1:**
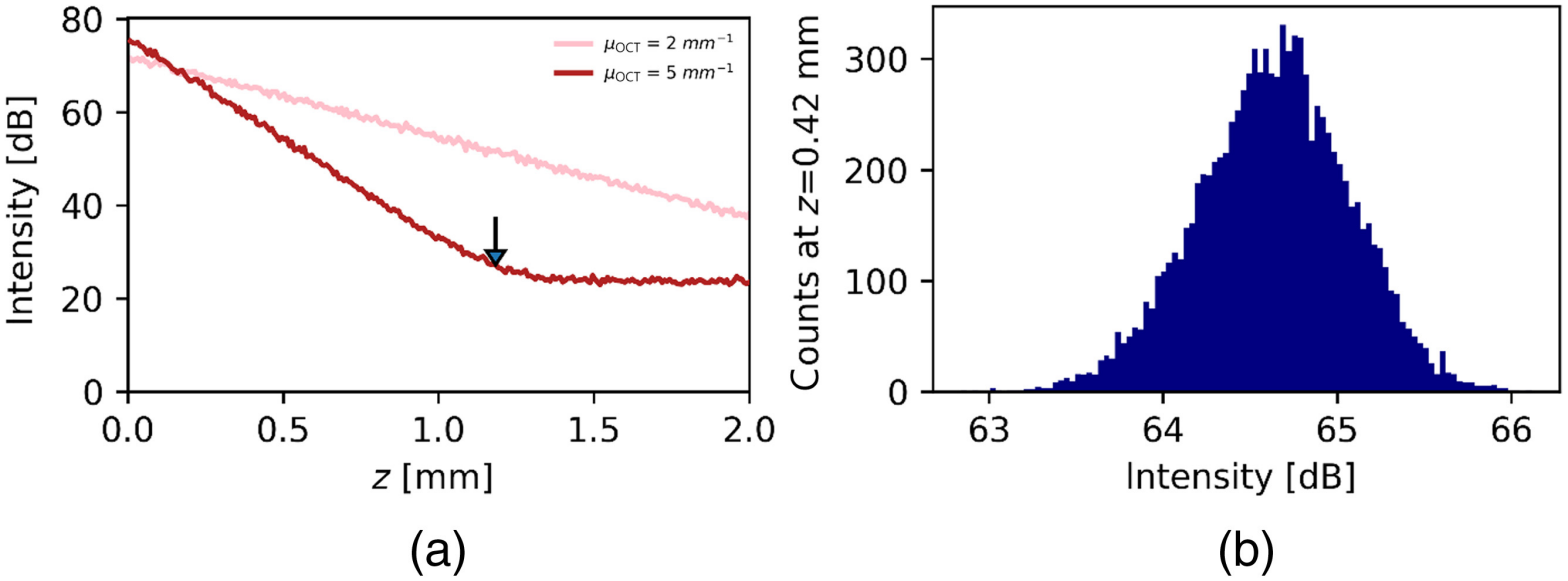
(a) Example of simulated, N=100 times averaged A-scans including shot noise (ζ=13.5) with $\mu_{\mathrm{OCT}}=2$ and $5{\;\mathrm{mm}}^{-1}$ using the model in Eq. (3). The backscatter efficiency within the NA $p_{\mathrm{NA}}$ was set to unity. The arrow indicates the position $z_{c}=1.18\;\mathrm{mm}$, where the OCT signal hits the noise floor. (b) The distribution of intensity values at z=0.42  mm obtained from $10^{4}$ independent simulations for $\mu_{\mathrm{OCT}}=2{\;\mathrm{mm}}^{-1}$.

Figure 1(a) shows an example of simulated N=100 times averaged A-scans, including shot noise, obtained by the procedure outlined in the previous section. The arrow indicates the position $z_{c}=1.18\;\mathrm{mm}$, at which the intensity signal hits the noise floor with the condition I[i]=⟨ζ⟩ for the simulation with $\mu_{\mathrm{OCT}}=5\;{\mathrm{mm}}^{-1}$ as input parameter. For $\mu_{\mathrm{OCT}}=2\;{\mathrm{mm}}^{-1}$ the intensity signal does not reach the noise floor.

Figure 1(b) shows a histogram of intensity values at a depth of z=0.42  mm using $\mu_{\mathrm{OCT}}=2\;{\mathrm{mm}}^{-1}$ obtained from of $10^{4}$ independent simulations. It shows that the averaged intensities, obtained by pre-averaging N=100 A-scans, are indeed to good approximation normally distributed.

**Fig. 2:**
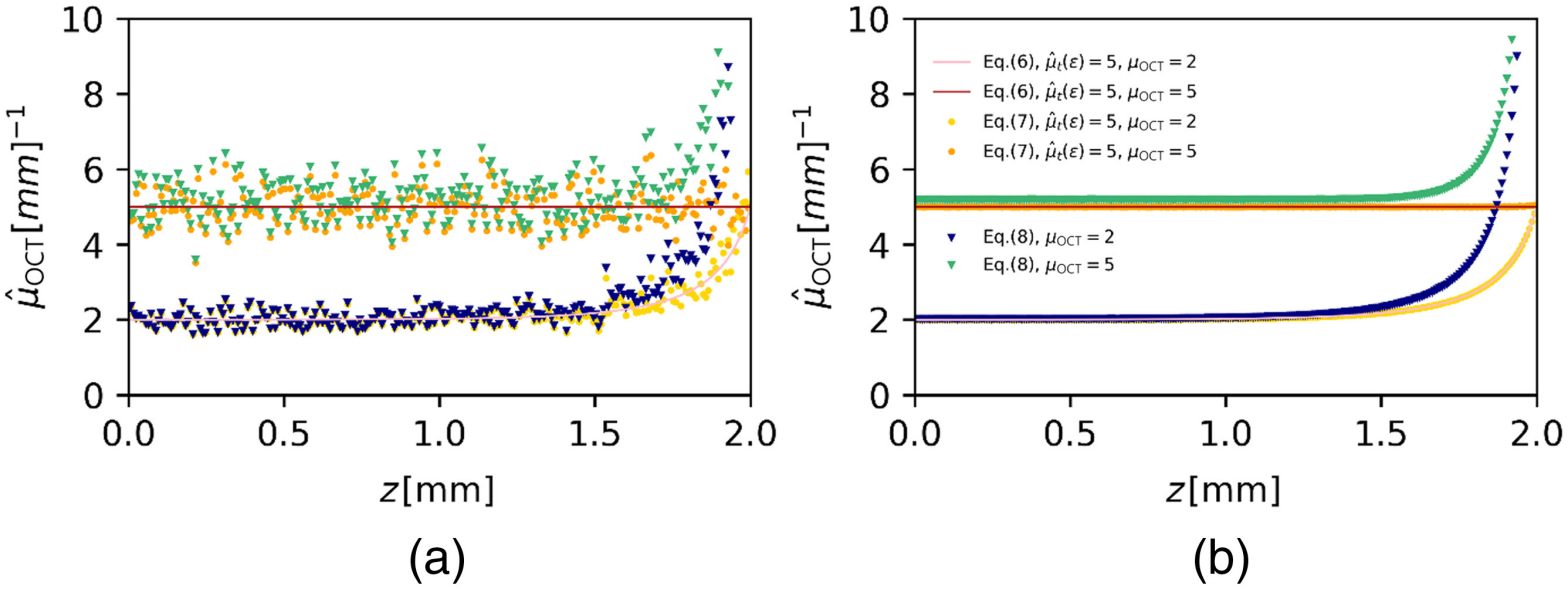
Accuracy of μ^OCT determination using the DRE method in absence of shot noise (ζ=0). Attenuation coefficients of $\mu_{\mathrm{OCT}}=2$ and $5{\;\mathrm{mm}}^{-1}$ were used to simulate N=100 times averaged A-scans according to the model of Eq. (3) with $p_{\mathrm{NA}}$ set to unity. The number of sample points is M=1, as the reconstruction is done per pixel. (a) Data points, show μ^OCT determined using the exact, discretized Eq. (7) and using its linearized approximation Eq. (8), $C_{L}=0$. Solid lines show the theoretical prediction of ⟨μ^OCT⟩ using Eq. (6). The mean per pixel of $10^{4}$ independent, averaged A-scans is compared to the theoretical value in (b).

**Fig. 3:**
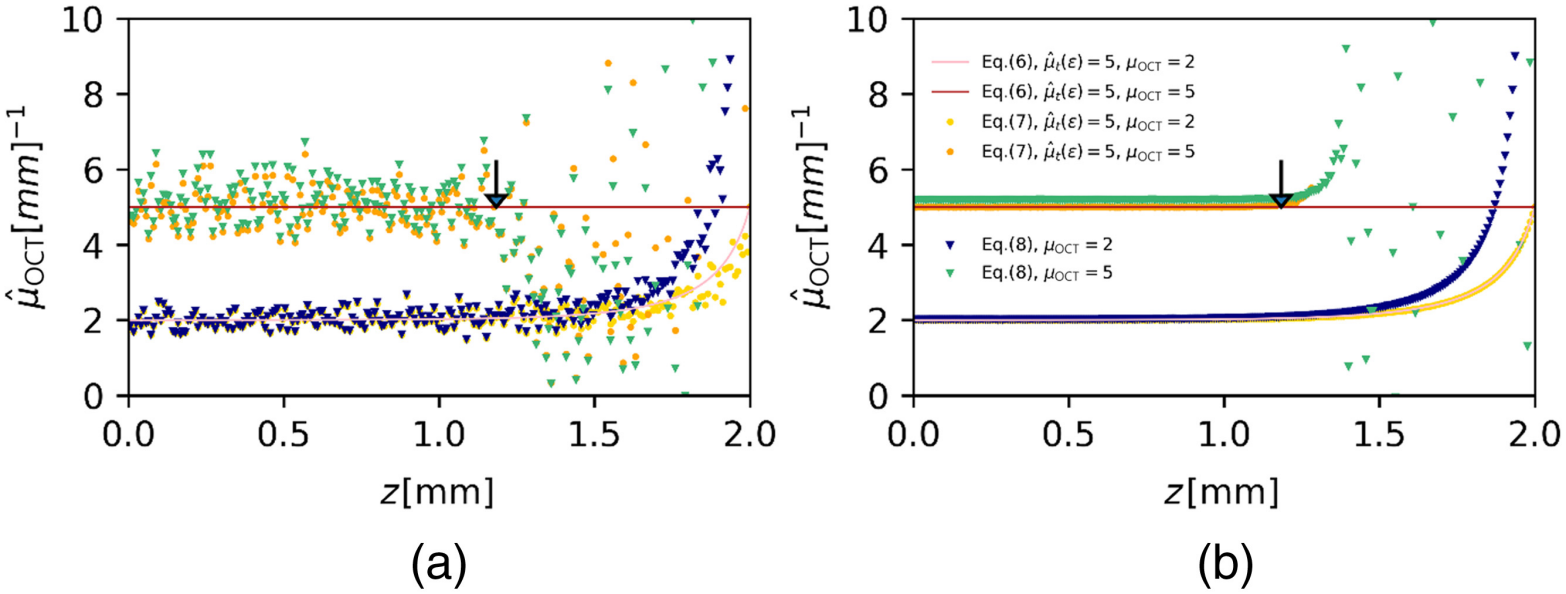
Accuracy of μ^OCT determination using the DRE method in the presence of shot noise (ζ=13.5). Attenuation coefficients of $\mu_{\mathrm{OCT}}=2$ and $5{\;\mathrm{mm}}^{-1}$ were used to simulate N=100 times averaged A-scans according to the model of Eq. (3) with $p_{\mathrm{NA}}$ set to unity. The number of sample points is M=1, as the reconstruction is done per pixel. The arrow indicates the depth position $z_{c}=1.18\;\mathrm{mm}$ where the OCT signal with $\mu_{\mathrm{OCT}}=5{\;\mathrm{mm}}^{-1}$ hits the noise floor. (a) Data points, shows μ^OCT, calculated using the exact, discretized Eq. (7), and using its linearized approximation Eq. (8), $C_{L}=0$. Solid lines show the theoretical prediction of ⟨μ^OCT⟩ using Eq. (6). The mean per pixel of $10^{4}$ independent, averaged A-scans is compared with the theoretical value in (b).

The assessment of the accuracy of the DRE method is shown in Fig. 2, in the absence of shot noise, and in Fig. 3, in the presence of shot noise for attenuation coefficients of 2 and $5\;{\mathrm{mm}}^{-1}$. Both figures show the estimated attenuation coefficients versus depth using Eqs. (7) and (8) with $C_{L}=0$ and the theoretical prediction of Eq. (6). Figure. 3(a) shows the DRE algorithm applied to a single averaged A-scan and demonstrates the remaining fluctuation in the estimations of $\mu_{\mathrm{OCT}}$; even after pre-averaging N=100 A-scans. The data shown in Fig. 3(b) is averaged over $10^{4}$ independent simulations and therefore permits closer comparison of theory and simulations. For the data shown in both figures, a value of μ^E=5  mm−1 is used in the regularization term C in Eq. (7). The results in Fig. 2(b) demonstrate that the estimated attenuation coefficient differs <1% from the true value up to a depth of 1.98 mm for $\mu_{\mathrm{OCT}}=2\;{\mathrm{mm}}^{-1}$ and, for $\mu_{\mathrm{OCT}}=5\;{\mathrm{mm}}^{-1}$, over the entire depth range. It approaches, in both cases, μ^E near the end of the available data range. If, on the other hand, the linearized approximation Eq. (8) with $C_{L}$ omitted is used, the attenuation coefficient has a fixed offset with respect to the true value and does therefore not stay within the 1% mark and, furthermore, tends to infinity at the end of the data range.

The more realistic case, when noise is included, is depicted in Fig. 3. Analogously to Fig. 2, the attenuation coefficients, calculated from one single, averaged A-scan (a) and their means per pixel (b) from $10^{4}$ independent estimations is shown. Including noise into the calculation results into a strong fluctuation of the estimated attenuation coefficient as soon as the signal hits the noise floor at $z_{C}$. This trend is expected as a result of using the full depth range for the attenuation coefficient estimation. The sum term in the denominator of Eqs. (7) and (8) is padded by random noise values fluctuating around zero after $z_{c}$ (assuming the average noise floor is correctly subtracted). The contribution of noise to the sum term and therefore the effect of including the noise region into the calculation increases with depth, as is clearly seen more clearly in Fig. 3(b) for depths larger than $z_{c}$. However, including the noise area into the calculation does not show a significant effect the attenuation estimation in the depth region before the signal hits the noise floor and differs <1% of true attenuation value up to a depth of 1.18 mm for $\mu_{\mathrm{OCT}}=5\;{\mathrm{mm}}^{-1}$ and, for $\mu_{\mathrm{OCT}}=2\;{\mathrm{mm}}^{-1}$, over the entire depth range similar to the results in Fig. 2.

**Fig. 4:**
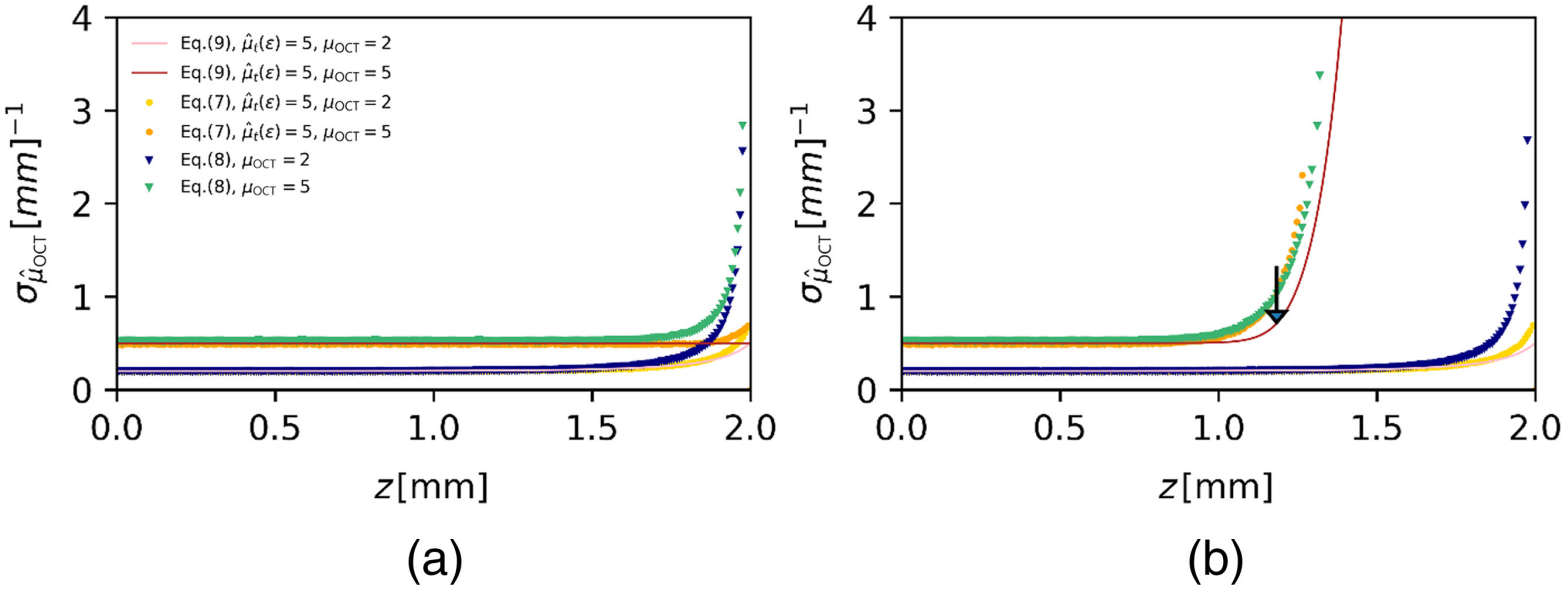
Precision of μ^OCT determination using the DRE method (a) in the absence (ζ=0). and (b) in the presence of shot noise (ζ=13.5). Attenuation coefficients of $\mu_{\mathrm{OCT}}=2$ and $5{\;\mathrm{mm}}^{-1}$ were used to simulate N=100 times averaged A-scans according to the model of Eq. (3) with $p_{\mathrm{NA}}$ set to unity. The number of sample points is M=1, as the reconstruction is done per pixel. The lower bound on the precision σμ^OCT,DRE (solid lines) was obtained from Eq. (9) and compared to the standard deviation σμ^OCT, (data points) obtained using DRE estimation based on the exact, discretized Eq. (7) and its linearized approximation [Eq. (8), $C_{L}=0$]. The arrow in (b) indicates the depth position $z_{c}=1.18\;\mathrm{mm}$ where the OCT signal with $\mu_{\mathrm{OCT}}=5{\;\mathrm{mm}}^{-1}$ hits the noise floor.

We proceed to compare the standard deviation σμ^OCT of the distribution of estimated attenuation coefficients at each depth with calculations based on Eq. (9) in Fig. 4(a) (without added shot nose) and Fig. 4(b) (with shot noise). It is shown in Fig. 4(a) that the results obtained using both the exact Eq. (7) and the approximation Eq. (8) with $C_{L}=0$ are in good agreement with the predictions of Eq. (9) except very near to the end of the available data range. Since by Eq. (9), the precision is proportional to the mean estimated attenuation coefficient at each depth [e.g., the results of Figs. 3(b) and 4(b)], it is found that the precision is slightly higher when Eq. (8) is used instead of Eq. (7). When shot noise is present [Fig. 4(b)], there is good qualitative agreement between the simulations and the predictions of Eq. (9) with the largest deviations occurring at depths where the signal is close to the noise floor.

**Fig. 5:**
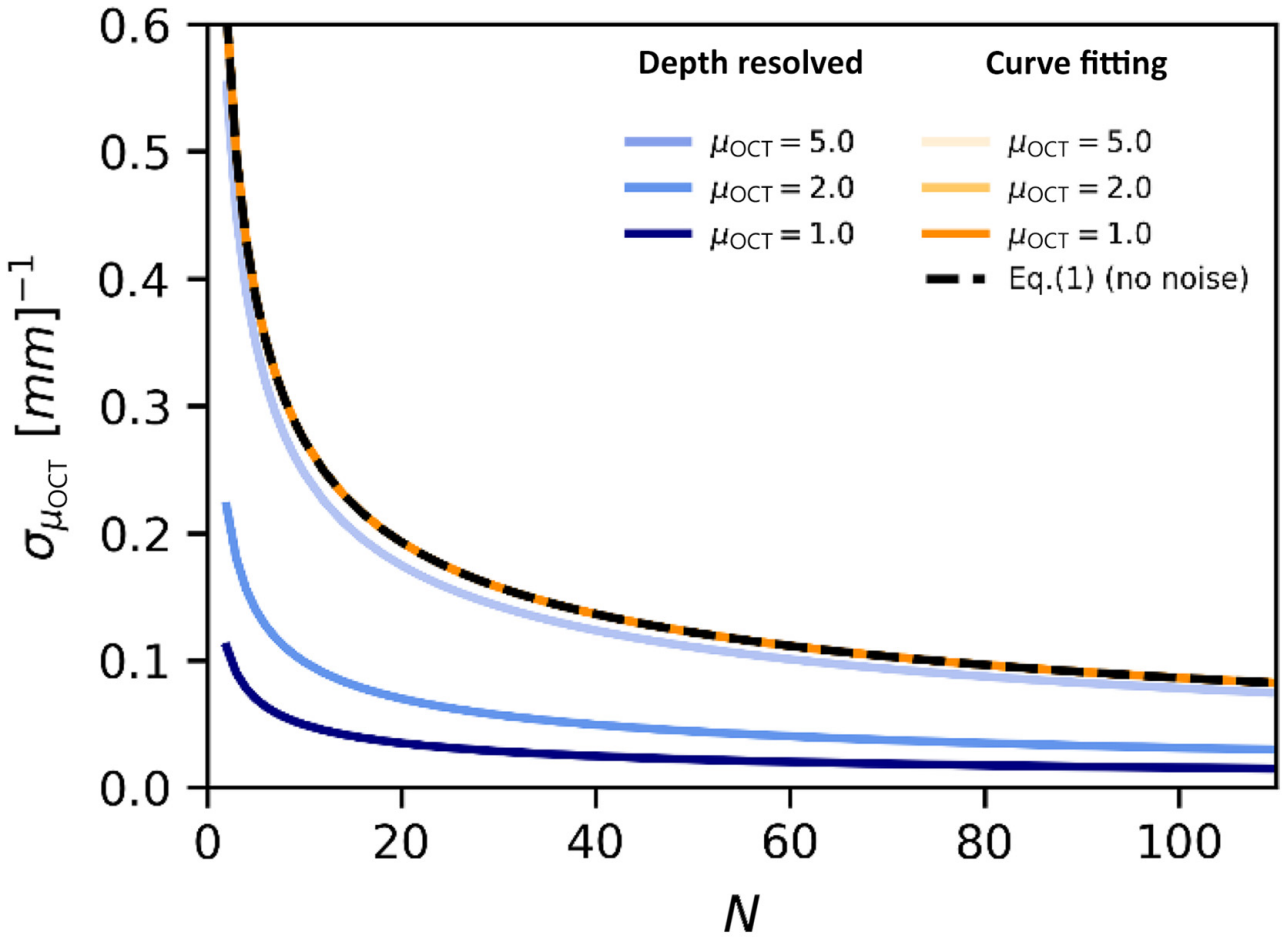
Comparison between the lower bound of the precision of the attenuation coefficient based on the DRE [Eq. (9)] and CF^7^ methods. The precision is shown in dependency on the number of averages N. Shot noise was included in the calculations (ζ=13.5). A region of interest from $z_{\min}=0.04\;\mathrm{mm}$ to $z_{\max}=0.36\;\mathrm{mm}$, with a length of 328  μm and M=41 sample points (step size Δ=8  μm), was used in both methods. The end-of-range values μ^E used in the DRE method were set to the true value of the attenuation coefficient $\mu_{\mathrm{OCT}}$. Note that the curves for the CF method overlap in this high-SNR limit.

Finally, we compared our previously reported lower bounds for the CF method^7^ with the precision we derived in this article for the DRE method in the presence of shot noise (ζ=13.5). Figure 5 shows the numerically obtained CR lower bound (the minimal precision for the CF approach) in dependence of N for an AFR of 328  μm (M=41 points, Δ=8  μm) located well before $z_{c}$ such that the SNR in the AFR is >20  dB. The black dashed line represents the analytical CRLB calculated using Eq. (1) and overlaps with the numerically obtained curves, thus demonstrating the validity of Eq. (1) for low noise levels as well as the independence of the lower bound on the value of the attenuation coefficient itself. The precision of the DRE method calculated by Eq. (9) (also using M=41 points, with end-of-range values μ^E set to the true value of $\mu_{\mathrm{OCT}}$.) does show a dependence on the attenuation coefficient. All curves follow a $1/\sqrt{N}$ trend, whereas σμ^OCT for the DRE method is smaller than for the CF method. Inspection of Eqs. (1) and (9) in the low-noise limit quickly reveals that the DRE method outperforms the CF method in precision, when $\mu_{\mathrm{OCT}}\cdot |\mathrm{AFR}|\leq \sqrt{3\cdot c_{R}}\approx 1.8$.

## 5 Discussion

Quantification of the attenuation coefficient requires thorough assessment of the accuracy and precision with which it can be estimated from OCT data. In recent years, the DRE method has emerged as an attractive alternative to the conventional approach of CF. We have derived expressions for the accuracy and precision of attenuation coefficient determined by the DRE method and validated those with numerical simulations.

The accuracy of the DRE method is given by Eq. (6). This equation includes a regularization term that sets the attenuation coefficient μ^E at the end of the available data range (either at the end of the A-scan, or the part of the A-scan that is included in the analysis). Omitting the regularization term essentially sets it value to infinity. This will result in an inaccurate attenuation estimation at the end of the data range, which can be overcome by choosing a region for attenuation reconstruction, which is far from the end of the data range. However, an accurate estimation can only be achieved when the attenuation coefficient is estimated using Eq. (7), which properly takes into account discretization of the OCT signal, as shown in Figs. 2 and 3. The approximate expression Eq. (8), which is often found in the literature, leads to a consistent overestimation of the attenuation coefficient in the order $\mu_{\mathrm{OCT}}^{2}\times \Delta$, where Δ is the pixel increment, and for that reason its use is highly discouraged. Next to inappropriate use of Eq. (8) [rather than Eq. (7)] that leads to a systemic offset, other factors that may contribute to a loss of accuracy are inadequate noise subtraction^16^ or incomplete compensation of the point spread function and roll-off function.^19^ Incorrect estimation of μ^E clearly leads to loss of accuracy at the end of the data range.

The expression for precision [Eq. (9), Appendix C] was derived under the premise that a number of N A-scans are averaged prior to application of the DRE method. Averaging results in approximately normally distributed averaged intensity values, which is the underlying assumption of the derivation in Appendix C. In the absence of noise, this leads to a CR lower bound on the precision for the estimation of the mean attenuation coefficient as σμOCT^=⟨μ^OCT⟩/MN. Contrary to our own advice in the previous paragraph, we continued to employ the linearized Eq. (8) with the purpose of arriving at a compact expression for the precision in the presence of noise. As evidenced from the results in Fig. 4(b), the resulting Eq. (9) is in good qualitative agreement with the simulation data and captures the effect of increasing SNR on the precision of the estimated attenuation coefficient. The key feature of Eq. (9) is that the precision depends on the mean estimated attenuation coefficient itself. Therefore, any loss in accuracy will directly lead to a loss of precision.

The dependence of precision on ⟨μ^OCT⟩ is also the main difference between the precision obtained through NLLS CF with two free running parameters, as expressed in Eq. (1), which depends on the extent of the AFR.^7^ Comparing both methods (Fig. 5), we see that the DRE method can obtain a better precision when $\mu_{\mathrm{OCT}}|\mathrm{AFR}|≲1.8$ when the same number of A-scans (N) is pre-averaged, and the same number of independent data points (M) is included in the analysis. Both methods thus require spatial support to achieve sufficient precision (the required level of precision may well depend on the application). In this sense, the term “depth resolved estimation” is somewhat misleading because in practice information from some spatial region must be included for the analysis.

### 5.1 Limitations

Simulations can be performed quickly and at low cost compared to the time and resources required for phantom experiments. Manufacturing of phantoms with precise control of the scattering properties can be challenging whereas simulations can explore a much wider range of parameter space beyond what is feasible in experiments. Simulations allow for precise control and manipulation of individual parameters (or tuning correlation among them), leading to deeper understanding of the underlying mechanisms. Although we have included only a limited number of $\mu_{\mathrm{OCT}}$ values and SNRs in the present article, our simulations are straightforwardly extended to include a wider range of scattering and absorption coefficients; to add point spread function and sensitivity roll-off [Eq. (2)] and introduce layers with their specific optical properties.

Performance of both the CF and DRE methods depends on the appropriateness of the underlying single-exponential decay model of Eq. (3) to describe the light–tissue interaction (assuming instrumental factors are corrected appropriately). In the this study, both the simulation generating OCT data and analysis were based on the same Eq. (3). Therefore, the accuracy and precision derived in this article represent the best values that can be obtained. This best-case scenario may not be true for experimental data, for instance when a small fraction of multiple scattering occurs. Multiple scattering models are available^20^ and can be adapted for CF, leading to the inclusion of one or more fit parameters describing tissue scattering (e.g., the root mean square scattering angle or scattering anisotropy). However, adaptation of these models for use in DRE seems challenging. In practice, even in the presence of multiple scattering, the part of the signal decay caused by absorption and scattering is often adequately modeled as a single exponential decay, albeit with a decay constant $\mu_{\mathrm{OCT}}\leq \mu_{s}+\mu_{a}$ (because multiple scattering causes more light to be detected, than expected based on the single-scattering model). Thus, the adoption of $\mu_{\mathrm{OCT}}$ allows us to describe tissue attenuation as measured by OCT as an effective parameter that does not require an estimate of the relative weight of single and multiple scattering contributions. When applying the DRE method to multiple layers with varying optical properties, the DRE method generally fails to extract the correct optical properties, unless for each layer $\mu_{\mathrm{OCT}}\propto \mu_{s}$ only (e.g., no absorption) and $p_{\mathrm{NA}}$ is a constant throughout the sample.^21^ Whether or not these conditions are met in practice should ultimately be verified by experiments while the level of inaccuracy and imprecision may be estimated using simulations.

### 5.2 Clinical Implications

Measurements of the attenuation coefficient complement the structural images that OCT provides. The main premise is that quantification of $\mu_{\mathrm{OCT}}$ can be used to distinguish different tissue types (e.g., benign versus malignant). Ideally, with perfect accuracy and precision, the sensitivity and specificity of such an approach are determined by the degree of biological variation within, and between the different tissue types. In practice, however, the accuracy and precision will be finite. The results laid out in this article, as well is in the previous publication^7^ allow us to determine to which degree the observed variation in attenuation coefficients is due to the employed method, and which part can be attributed to biological variation.

## 6 Conclusion

In this article, we derived and validated the accuracy and precision of the depth resolved estimation method of the attenuation coefficient $\mu_{\mathrm{OCT}}$ in optical coherence tomography. We showed that a commonly used simplification of the method results in loss of accuracy in the order of $\Delta \times \mu_{\mathrm{OCT}}^{2}$ where Δ is the sampling resolution and is therefore not recommended as being used for OCT-attenuation reconstruction. Furthermore, we derived an analytical expression for the precision of μ^OCT, which proportionally scales with its expectation value and inversely with the square root of the number of independent sample points included in the analysis. Lastly, we compared our outcome with the precision obtained using a CF procedure and provided an easy applicable rule of thumb to determine which method will have a better precision. Our theoretical framework gives valuable insight regarding accuracy and precision of parametric imaging based on a depth-resolved reconstruction of the attenuation coefficient and is, given its wide and easy-to-use applicability, an important advance toward design and improvement of standardized OCT-experiments, which are, e.g., used for tissue characterization in the clinic.

## 7 Appendix A

We model the mean OCT signal intensity as function of depth in Eq. (3) using a single exponential decay function, assuming that the confocal point spread function and the sensitivity roll-off are fully compensated and that a constant mean noise floor is subtracted. We first compute the integral $I_{\infty }(z)=-\int_{z}^{\infty }I(z^{\prime })dz^{\prime }$ of Eq. (3), which yields

$$I_{\infty }(z)=\frac{p_{\mathrm{NA}}\mu_{s}}{2\mu_{\mathrm{OCT}}}\;\exp(-2\mu_{\mathrm{OCT}}z).$$ (10)

Taken together with Eq. (3), we can solve for the attenuation coefficient as

$$\mu_{\mathrm{OCT}}(z)=\frac{I(z)}{2I_{\infty }(z)}.$$ (11)

In practice, data are only available over a finite range, up to z=E. We compute the definite integral $I_{E}(z)=-\int_{z}^{E}I(z^{\prime })dz^{\prime }$, which yields

$$I_{E}(z)=\frac{p_{\mathrm{NA}}\mu_{s}}{2\mu_{OCT}}[\exp(-2\mu_{\mathrm{OCT}}z)-\exp(-2\mu_{\mathrm{OCT}}E)]=I_{\infty }(z)-\frac{I(E)}{2\mu_{\mathrm{OCT}}}.$$ (12)

Solving Eq. (12) for $I_{\infty }(z)$, and substituting the result in Eq. (11) gives

$$\mu_{\mathrm{OCT}}=\frac{I(z)}{2I_{E}(z)+\frac{I(E)}{\mu_{\mathrm{OCT}}}}.$$ (13)

Finally, Eq. (13) can be rearranged to solve for the attenuation coefficient giving the finite-range equivalent of Eq. (11)

$$\mu_{\mathrm{OCT}}(z)=\frac{I(z)-I(E)}{2I_{E}(z)}.$$ (14)

When z approaches the end of range E, the finite integral in the denominator term goes to zero; therefore $2I_{E}(z)\to 0$ and the estimation of the attenuation coefficient in Eq. (14) will tend to infinity. A better strategy is to regularize the depth resolved estimation using an independently obtained estimate for the value of the attenuation coefficient at the end of the data range,^12^ μ^E=μOCT(E). This allows us to rewrite Eq. (13) as an estimator of $\mu_{\mathrm{OCT}}$:

μ^OCT(z)=I(z)2IE(z)+I(E)μ^E=μOCT1−[1−μOCTμ^E]exp(−2μOCT(E−z)). (15)

Equation (15) reveals that the estimate μ^OCT(z) approaches the true value $\mu_{\mathrm{OCT}}$ at a z-position sufficiently far from the end of range E, whereas μ^OCT(z) approaches the estimate μ^E as z approaches E.

## 8 Appendix B

Vermeer et al.^9^ considered the effect of discretization of I(z). Each data point I[i] corresponds to the integration of Eq. (3) over a finite pixel size Δ around z. They show that the discretized version of Eq. (11) reads

μ^OCT[i]=12Δ ln(1+I[i]∑j=i+1∞I[j]), (16)

where μ^OCT[i] is now the estimate of the average attenuation coefficient in the i’th pixel. Considering that data are only available over a finite data range we write

μ^OCT[i]=12Δ ln(1+I[i]∑j=i+1imaxI[j]+C), (17)

where $i_{\max}$ is the pixel index corresponding to the end of range E and $C=\overset{\infty }{\underset{j=i_{\max}+1}{\sum }}I[j]$.

We can use Eq. (16) to obtain an expression for C. We have μ^OCT[imax]=12Δ ln(1+I[imax]∑j=imax+1∞I[j])=12Δ ln(1+I[imax]C) so that $C=I[i_{\max}]/(\exp(2\mu_{E}\Delta )-1)$. Here, $\mu_{E}=\mu_{\mathrm{OCT}}[i_{\max}]$ is an independent estimate of the attenuation coefficient at the end of the range, as before (Appendix A).

Quite often, approximate forms of Eq. (7) / Eq. (17) are found in literature, which are obtained by linearization of the logarithmic and exponential terms. Then, factor $C\approx I[i_{\max}]/2\mu_{E}\Delta$ and upon expanding the logarithmic term $\ln(1+x)=x-\frac{1}{2}x^{2}+\ldots$:

μ^OCT[i]=I[i]2Δ∑j=i+1imaxI[j]+I[imax]μE−Δ(I[i]2Δ∑j=i+1imaxI[j]+I[imax]μE)2+…, (18)

after which only the first term is retained. Under that same approximation, the second term in Eq. (18) is approximately equal to $\mu_{\mathrm{OCT}}^{2}\times \Delta$ and we conclude that linearization of Eq. (7)/(17) leads to a systematic overestimation of the attenuation coefficient in the order of $\mu_{\mathrm{OCT}}^{2}\times \Delta$.

## 9 Appendix C

We seek the precision of the DRE method in the presence of noise. We make use of the fact that the attenuation coefficient will be approximately normally distributed, and that the precision is given by the standard deviation σμ^OCT of that distribution. We use the “simplified” form of the depth resolved method

μ^OCT(i)=I(i)2Δ(∑j=i+1MI(j)+C). (19)

After pre-averaging N>30 times, the intensity values are normally distributed. The term $D(i)=\overset{M}{\underset{j=i+1}{\sum }}I(j)+C$ in the denominator is then the sum of normally distributed random variables plus a constant, which yields a new normal variable with mean $m_{D}(i)=\overset{M}{\underset{j=i+1}{\sum }}m_{I}(j)+C$ and variance $\sigma_{D}^{2}(i)=\sum_{j=i+1}^{M}[\sigma_{I}^{2}(j)+\sigma_{\varsigma }^{2}]$; that is, the means and variances simply add up. When the coefficients of variation of either the nominator $\delta_{I}=\sigma_{I}/m_{I}$ or the denominator $\delta_{D}=\sigma_{D}/m_{D}$ in the ratio is <1 (in fact, both are), the result is also normally distributed with mean

mμ^OCT(i)=mI2ΔmD(i)≡⟨μ^OCT(i)⟩. (20)

The variance is given as

σμ^OCT2(i)=(mI2ΔmD(i))2(δI2+δD2). (21)

Fiske et al.^18^ showed that the coefficient of variation $\delta_{D}\ll 1$ already, even without pre-averaging so we neglect that term in Eq. (21). See also Appendix D for further justification.

Thus, upon averaging N>30 of individual A-scans followed by subtraction of the mean noise level $\left\langle \varsigma_{I}\right\rangle$ [Eq. (2)], the signal I(i) is obtained, mean $m_{I}\equiv \langle I(i)\rangle$ and variance $\sigma_{I}^{2}=(\frac{{\left\langle I(i)\right\rangle }^{2}}{N}+\frac{{\left\langle \varsigma_{I}\right\rangle }^{2}}{N})=(\frac{m_{I}^{2}}{N}+\frac{{\left\langle \varsigma_{I}\right\rangle }^{2}}{N})$. These relations indicate that subtracting the mean noise floor does not remove the fluctuations caused by noise. The required expression for $\delta_{I}^{2}=\sigma_{I}^{2}/m_{I}^{2}$ (the square of the coefficient of variation CV) is

$$\delta_{I}^{2}(i)=\frac{1}{N}(1+\frac{{\left\langle \varsigma_{I}\right\rangle }^{2}}{m_{I}^{2}(i)})=\frac{1}{N}(1+\frac{1}{\mathrm{SNR}{\left(i\right)}^{2}}),$$ (22)

where the SNR is now defined as $\mathrm{SNR}=m_{I}/\langle \varsigma_{I}\rangle$. Combining with Eq. (21), assuming M independent estimations, the precision expressed as standard deviation becomes

σμ^OCT(i)=mμ^OCT(i)MN1+1SNR(i)2. (23)

## 10 Appendix D

Let $D(i)=\sum_{j=i+1}^{M}I(j)+C$ be the sum term in the denominator of Eq. (19). It is the sum of normally distributed random variables plus a constant, which yields a new normal variable with mean $m_{D}(i)=\overset{M}{\underset{j=i+1}{\sum }}m_{I}(j)+C$ and variance $\sigma_{D}^{2}(i)=\sum_{j=i+1}^{M}[\sigma_{I}^{2}(j)+\sigma_{\varsigma }^{2}]$; that is, the means and variances simply add up. The square of the coefficient of variation is

$$\delta_{D}^{2}(i)=\frac{\sigma_{D}^{2}(i)}{(m_{D}(i){)}^{2}}=\frac{\sum_{j=i+1}^{M}[\sigma_{I}^{2}(j)+\sigma_{\varsigma }^{2}]}{{\left(\sum_{j=i+1}^{M}m_{I}(j)+C\right)}^{2}}=\frac{1}{N}\{\frac{\sum_{j=i+1}^{M}[m_{I}^{2}(j)+{\left\langle \varsigma_{I}\right\rangle }^{2}]}{{\left(\sum_{j=i+1}^{M}m_{I}(j)+C\right)}^{2}}\}.$$ (24)

We will first work out the term without noise, then the term with noise, making use of the fact that the analysis is based on the exponential decay model so we can write inside the summations: $m_{I}(j)=m_{I}(i)\;\exp(-2\mu_{\mathrm{OCT}}\Delta \cdot (j-i))$.

Let k=j−i, $x=e^{-2\mu_{\mathrm{OCT}}\Delta }$ and use the identity:

$$\overset{M-i}{\underset{k=1}{\sum }}x^{k}=\frac{x^{1}-x^{M-i+1}}{1-x}=\frac{x}{1-x}(1-x^{M-i}).$$ (25)

Then, the mean of D(i) follows as

$$m_{D}(i)=\overset{M}{\underset{j=i+1}{\sum }}m_{I}(j)+C=\overset{j=M}{\underset{j=i+1}{\sum }}m_{I}(i)e^{-2\mu_{\mathrm{OCT}}\Delta \cdot (j-i)}+C\;\Rightarrow \overset{k=M-i}{\underset{k=1}{\sum }}m_{I}(i)e^{-2\mu_{\mathrm{OCT}}\Delta \cdot (k)}+C=m_{I}(i)\frac{e^{-2\mu_{\mathrm{OCT}}\Delta }}{1-e^{-2\mu_{\mathrm{OCT}}\Delta }}(1-e^{-2\mu_{\mathrm{OCT}}\Delta (M-i)})+C.$$ (26)

And the variance as

$$\sigma_{D}^{2}(i)=\frac{1}{N}\overset{M}{\underset{j=i+1}{\sum }}[m_{I}^{2}(j)+{\left\langle \varsigma_{I}\right\rangle }^{2}]\Rightarrow \sigma_{D}^{2}(i)=\frac{1}{N}\overset{M}{\underset{j=i+1}{\sum }}[m_{I}(i{)}^{2}e^{-4\mu_{\mathrm{OCT}}\Delta \cdot (j-i)}+{\left\langle \varsigma_{I}\right\rangle }^{2}]=\;\frac{1}{N}[m_{I}{\left(i\right)}^{2}\frac{e^{-4\mu_{\mathrm{OCT}}\Delta }}{1-e^{-4\mu_{\mathrm{OCT}}\Delta }}(1-e^{-4\mu_{\mathrm{OCT}}\Delta (M-i)})+(M-i){\left\langle \varsigma_{I}\right\rangle }^{2}].$$ (27)

Inserting the expressions for the SNR and regularization term C:

δD2(i)=1N[e−4μOCTΔ1−e−4μOCTΔ(1−e−4μOCTΔ(M−i))+(M−i)SNR2(e−2μOCTΔ1−e−2μOCTΔ(1−e−2μOCTΔ(M−i))+e−2μOCTΔ(M−i)(exp(2Δμ^E)−1))2]. (29)

To arrive at a more compact, albeit approximate expression we first linearize the exponentials

δD2(i)≈1N[(1−4μOCTΔ)(M−i)+(M−i)SNR2((1−2μOCTΔ)(M−i)+1−2μOCTΔ(M−i)2Δμ^E)2], (30)

δD2(i)≈1N[(1−4μOCTΔ)(M−i)+(M−i)SNR2((1−2μOCTΔ)(M−i)(1+12Δμ^E))2]. (31)

Then assume $\mu_{\mathrm{OCT}}\Delta \ll 1$ and rearrange to

δD2(i)≈1N(1+1SNR2)((2μ^EΔ)2M−i). (32)

Comparing to Eq. (22), we see that δD2(i)=δI2(i)×(2μ^EΔ)2/(M−i). Since (μ^E)−1 is in the order of mm and Δ is in the order of μm, neglecting $\delta_{D}^{2}(i)$ as is done in Appendix C is justified.

## Code, Data, and Materials Availability

The code and data are available from the authors upon request.
